# Retrospective efficacy analysis of stellate ganglion block combined with general anesthesia in arthroscopic shoulder surgery: a cohort study

**DOI:** 10.3389/fmed.2025.1625121

**Published:** 2025-09-15

**Authors:** Zilin Pan, Jiawei Li, Yizhen Xu, Yingchuan Yuan

**Affiliations:** Department of Anesthesiology, The Second Affiliated Hospital of Xinjiang Medical University, Ürümqi, Xinjiang, China

**Keywords:** stellate ganglion block, shoulder arthroscopy, perioperative stability, analgesia, recovery efficiency

## Abstract

**Purpose:**

Although arthroscopic shoulder surgery confers clear advantages for recovery, it is often complicated by intraoperative hemodynamic instability and prolonged postoperative pain. This study investigates the clinical utility of stellate ganglion block (SGB) combined with general anesthesia (GA) to address these issues.

**Methods:**

In this retrospective analysis, 60 patients undergoing elective shoulder arthroscopy were categorized into SGB+GA (*n* = 30) and GA-only (*n* = 30) cohorts. Outcomes included intraoperative hemodynamic parameters (MAP, HR), postoperative pain (VAS scores), opioid-related complications, and hospitalization duration. Statistical analyses utilized *t*-tests and non-parametric tests.

**Results:**

The SGB+GA group demonstrated superior hemodynamic stability (*P* < 0.001) and markedly lower pain scores postoperatively (*P* < 0.001). Opioid-induced complications such as nausea (*P* = 0.028) were significantly reduced. Median hospital stay was shorter with SGB+GA (*P* < 0.001).

**Conclusion:**

Integrating SGB with GA optimizes perioperative management by stabilizing hemodynamics, enhancing analgesia, and minimizing opioid reliance, thereby expediting recovery. These findings support SGB as a valuable adjunct, though prospective validation is needed.

## 1 Introduction

Arthroscopic shoulder surgery, recognized as the gold standard for managing shoulder pathologies, offers numerous advantages due to its minimally invasive approach and favorable recovery profile (1). Optimizing regional anesthesia for these procedures is crucial to ensure adequate analgesia while minimizing side effects associated with general anesthesia, such as sedation and postoperative nausea and vomiting (PONV), thereby enhancing postoperative recovery. However, significant challenges persist, including intraoperative hemodynamic instability and persistent postoperative pain linked to sympathetic hyperactivity and neuroinflammation (2, 3). Traditional peripheral nerve block techniques, notably interscalene brachial plexus block (ISB) (4) and suprascapular nerve block (SNB) (5), have limitations such as rebound pain upon block resolution,^1^ diaphragmatic paralysis, and inadequate control of sympathetically mediated pain. These limitations underscore the need for the development and implementation of more advanced or multifaceted analgesic strategies.

SGB provides a dual mechanism of action (6, 7): it stabilizes hemodynamics by suppressing sympathetic overactivity and reduces inflammatory mediators, thereby targeting both nociceptive and neuropathic pain pathways (8). Although SGB has been proven effective in thoracic and vascular surgeries, its synergistic effects with general anesthesia in shoulder arthroscopy remain underexplored. Notably, while interscalene brachial plexus block (ISB) is the current gold standard for perioperative analgesia in shoulder arthroscopy (4, 9), its implementation may be limited by resource constraints or contraindications [e.g., coagulopathy, pre-existing neuropathy, severe chronic obstructive pulmonary disease (COPD), obstructive sleep apnea (OSA) and obesity]. Thus, exploring SGB as an alternative remains clinically relevant. its synergistic effects with general anesthesia in shoulder arthroscopy remain underexplored, which limits evidence-based integration.

This trial evaluates the use of SGB in conjunction with general anesthesia during arthroscopic shoulder surgery. Our findings aim to refine perioperative protocols and improve clinical efficiency.

## 2 Materials and methods

### 2.1 General information

Ethical approval for this study was obtained from the Institutional Review Board of the Second Affiliated Hospital of Xinjiang Medical University (Approval No. LW2024092201). This program ensured the scientific, ethical, and compliant nature of the study and safeguarded the rights and safety of the participants. Initial screening identified 48 eligible patients. After comprehensive evaluation, 18 were excluded:

1. Preoperative exclusions (*n* = 12): ASA > II: uncontrolled hypertension (*n* = 3), diabetic nephropathy (*n* = 2), Chronic opioid use (> 30 MME/day, *n* = 3), Contraindications to local anesthetics: coagulopathy (INR > 1.5, *n* = 2), Prior ipsilateral shoulder surgery (*n* = 1), Cervical pathology affecting SGB (*n* = 1),
2. Intraoperative exclusions (*n* = 4): SGB failure (absence of Horner’s syndrome, *n* = 2), Vasoactive drug requirement (norepinephrine infusion, *n* = 2),
3. Postoperative data exclusions (*n* = 2): incomplete outcome records (> 20% missing VAS/PCA data, *n* = 2).

Thirty patients receiving SGB+GA, matched 1:1 based on anesthesia records from December 2020 to August 2023 to GA-only controls using criteria including age (±5 years), ASA status, and surgery duration (±15 min). Cohort assignment was based on documented anesthesia protocols. Rationale for GA-only control: this design was selected to (a) reflect real-world practice in settings lacking regional anesthesia expertise, and (b) establish baseline efficacy of SGB+GA before comparative studies with ISB. According to institutional records, 35% of shoulder arthroscopies during 2020–2023 received GA-only due to contraindications to regional techniques or anesthesiologist availability. Patients receiving SGB+GA were classified as the intervention cohort (SGB+GA, *n* = 30), while those receiving GA-only comprised the control cohort (GA-only, *n* = 30). As a retrospective analysis of existing data, this study did not require prospective trial registration.

The baseline characteristics, including age, gender, ASA classification, and operative duration, exhibited no significant intergroup differences (*P* > 0.05), thereby ensuring comparability.

### 2.2 Intervention protocols

#### 2.2.1 Group SGB+GA

SGB procedure: SGB was performed under real-time ultrasound guidance with color Doppler, using a high-frequency linear probe in a transverse short-axis view of the neck. The target injection site was identified anterior to the longus colli muscle and posterior to the carotid sheath at the level of the C6 transverse process. A paracarotid lateral approach was selected, and the needle trajectory was planned under Doppler guidance to avoid aberrant vessels such as the inferior thyroid artery and vertebral artery. The needle was advanced using an in-plane technique until the tip reached the target location. After confirming negative aspiration for blood or cerebrospinal fluid, 10 mL of 0.25% ropivacaine was injected (7). Successful blockade was confirmed by the development of ipsilateral Horner’s syndrome (miosis, ptosis, and anhidrosis) (10).

##### 2.2.1.1 General anesthesia

Induction: Administer midazolam (0.05 mg/kg), propofol (1.5–2.0 mg/kg), sufentanil (0.4–0.5 μg/kg), and cisatracurium (0.15–0.20 mg/kg), followed by tracheal intubation.

Maintenance: Administer propofol (4–8 mg/kg/h) and remifentanil (0.1–0.2 μg/kg/min), adjusting dosages to maintain a Bispectral Index (BIS) of 40–60 and stable hemodynamics. As needed, sevoflurane, propofol, remifentanil, and rocuronium may be administered to ensure adequate depth of anesthesia.

#### 2.2.2 Group GA-only

Identical induction and maintenance protocols were implemented, excluding SGB.

Postoperative Pain Management (Both Groups):

All patients were given intravenous patient-controlled analgesia (PCA) following this protocol:

Sufentanil at a concentration of 2 μg/mL in a total volume of 100 mL.

Continuous infusion: 2 mL per hour.

Bolus dose: 2 mL per dose, with a lockout period of 5 min. For additional pain relief, intramuscular tramadol 50 mg was provided if the resting VAS score was 6 or higher.

Inclusion criteria:

1. Type: Patients undergoing elective arthroscopic shoulder surgery.
2. Age: 20 to 65 years.
3. Health status: ASA physical status I–II.
4. Anesthesia contraindication: No contraindications to local anesthetics.
5. Data completeness: Complete hemodynamic records, postoperative pain scores, and opioid consumption data.

Exclusion criteria:

1. Intraoperative exclusions:
  SGB failure (absence of Horner’s syndrome).
  Use of vasoactive drugs (e.g., norepinephrine).
2. Clinical factors:
  Severe cardiopulmonary/hepatic/renal dysfunction or coagulopathy.
  History of psychiatric/cognitive disorders.
  Prior ipsilateral shoulder surgery/trauma.
  Cervical spine pathology affecting SGB efficacy.
3. Data missingness: ≥ 20% missing key outcomes (MAP, HR, VAS).
4. Special populations: Pregnancy or lactation.

### 2.3 Outcome measures

#### 2.3.1 Primary outcomes

##### 2.3.1.1 Pain assessment

(1) Postoperative pain was quantified utilizing Visual Analog Scale (VAS) scores collected at 2, 6, 12, and 24 h following surgery.

(2) The overall usage of remifentanil during surgery and the total amount of tramadol used within 24 h after the operation.

#### 2.3.2 Secondary outcomes

(1) Intraoperative hemodynamics were evaluated through the measurement of blood pressure and heart rate at five designated time points: baseline (T0), post-induction (T1), during incision (T2), 30 min into surgery (T3), and at the procedure’s conclusion (T4).

(2) Potential adverse effects encompass nausea, vomiting, dizziness, drowsiness, and respiratory depression.

(3) The duration of hospital stay.

### 2.4 Statistical methods

Outcome assessors for VAS and complications were blinded to group allocation using anonymized patient identifiers. Data were analyzed using SPSS version 22.0. Continuous variables (MAP, HR, and VAS) are expressed as mean ± standard deviation (SD) and compared with independent *t*-tests. Categorical variables (e.g., complications) were analyzed using chi-square tests or Fisher’s exact tests, the latter applied when expected cell frequencies were < 5. A two-tailed *P*-value < 0.05 was considered statistically significant. Multivariate regression controlled for age/ASA status.

## 3 Results

### 3.1 General conditions

There were no significant differences between the two groups in terms of gender, age, ASA classification, operation duration, and intraoperative fluid after comparison (*P* > 0.05; see Table 1).

**TABLE 1:** Comparison of general condition of patients in 2 groups (*n* = 30).

Characteristic	Group		*p*-value
	GA-only, *N* = 30^1^	SGB+GA, N = 30^1^	
Age (years)	42 ± 7	43 ± 8	0.643^2^
Surgery duration (min)	117 (110, 125)	116 (103, 127)	0.584^3^
Intraoperative fluid (ml)	2,125 ± 165	2,136 ± 146	0.789^2^
**Sex (M/F)**			0.796^4^
F	16 (53.3%)	15 (50.0%)	
M	14 (46.7%)	15 (50.0%)	
**ASA I/II**			0.417^4^
I	21 (70.0%)	18 (60.0%)	
II	9 (30.0%)	12 (40.0%)	

^1^Mean ± SD; median (IQR); *n* (%).

^2^Welch two sample *t*-test.

^3^Wilcoxon rank sum test.

^4^Pearson’s chi-squared test.

### 3.2 Intraoperative hemodynamic indicators

**FIGURE 1:**
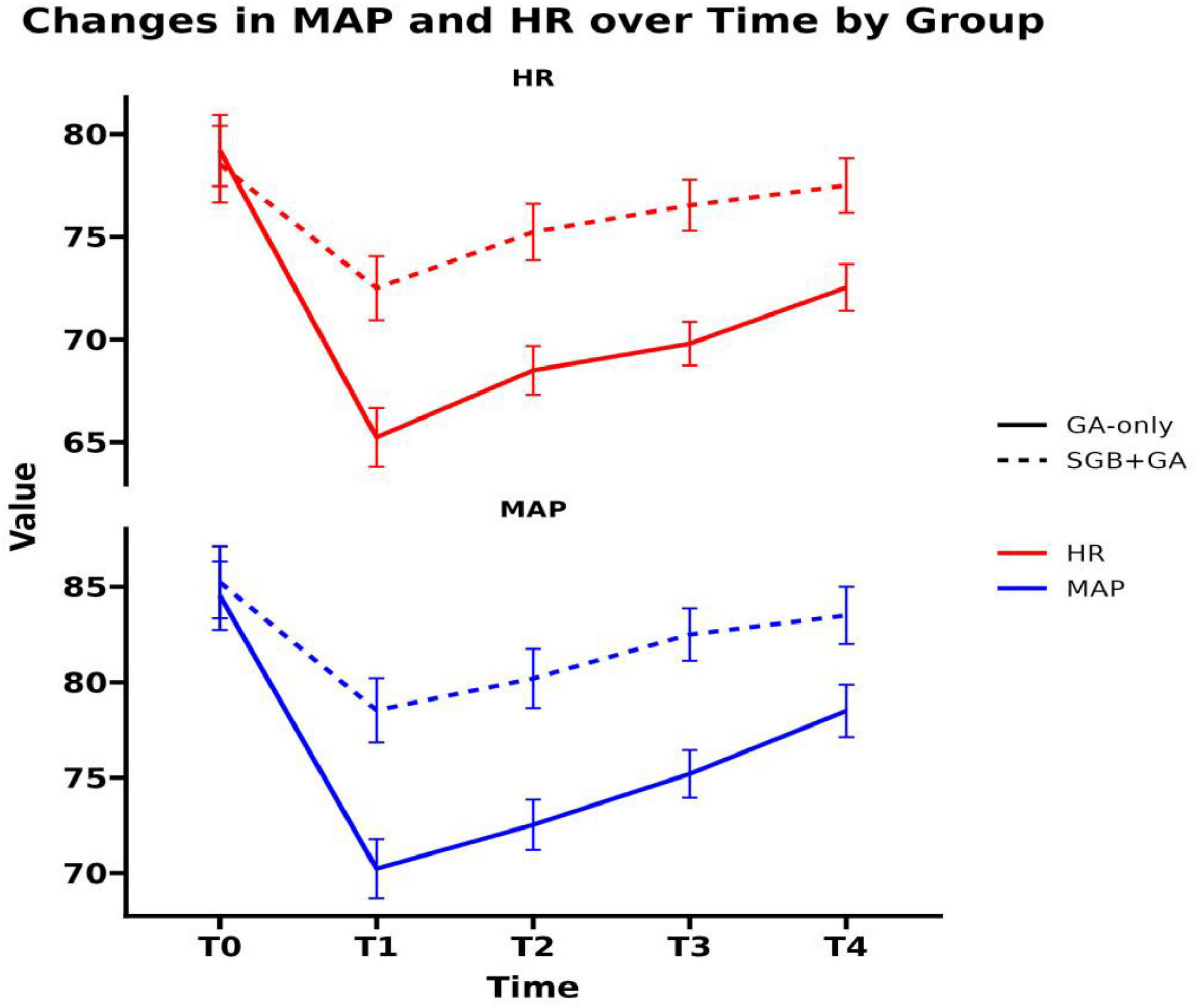
Vital signs at different time points.

At T0, there was no statistically significant difference in MAP and HR between the two groups (*P* > 0.05). The fluctuation amplitudes of MAP and HR in SGB+GA group from T1 to T4 were smaller than those in the control group (see Figure 1), and this difference was statistically significant (*P* < 0.05; see Table 2).

**TABLE 2:** Comparison of intraoperative hemodynamic indexes in 2 groups (***n*** = 30).

Characteristic	Group		*p*-value	Effect size
	GA-only, *N* = 30^1^	SGB+GA, *N* = 30^1^		
T0MAP	85 ± 10	85 ± 10	0.789^2^	0.04^4^
T0HR	79 ± 10	79 ± 10	0.795^2^	0.16^4^
T1MAP	70 (64, 76)	79 (72, 88)	0.001^3^	**1.09** ^4^
T1HR	64 (59, 73)	78 (61, 78)	< 0.001^3^	0.81^4^
T2MAP	73 ± 7	80 ± 9	< 0.001^2^	**0.91** ^4^
T2HR	69 ± 7	75 ± 8	< 0.001^2^	**0.97** ^4^
T3MAP	77 (71, 79)	87 (76, 88)	< 0.001^3^	0.75^4^
T3HR	69 (66, 74)	80 (73, 81)	< 0.001^3^	0.81^4^
T4MAP	79 ± 8	84 ± 8	0.017^2^	0.69^4^
T4HR	73 ± 6	78 ± 7	0.006^2^	0.55^4^

^1^Mean ± SD; median (IQR).

^2^Welch two sample *t*-test.

^3^Wilcoxon rank sum test.

^4^Cohen’s d. Bolded values indicate statistically significant differences (i.e., *P* < 0.05).

### 3.3 Postoperative pain scores

**FIGURE 2:**
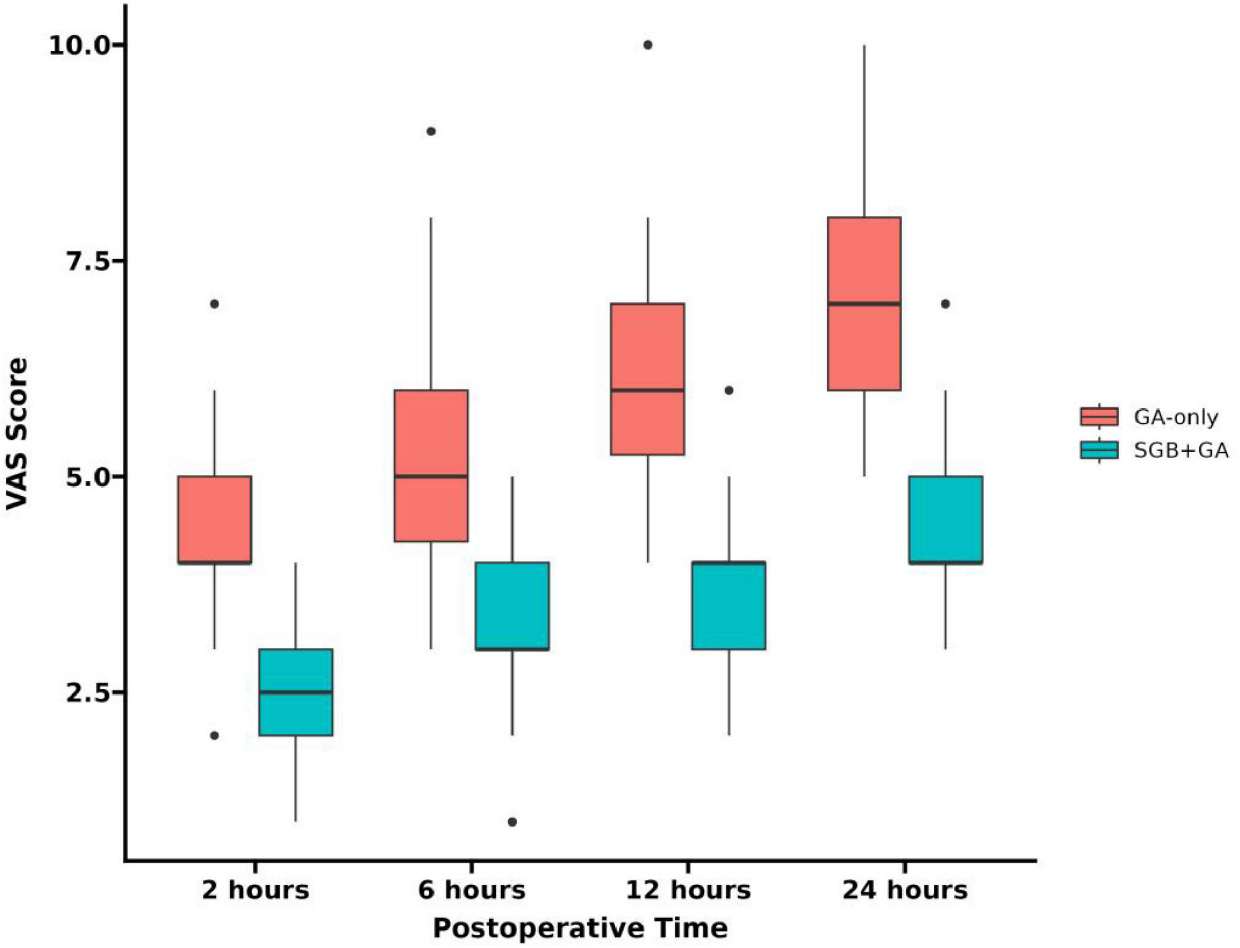
VAS scores at different time points.

The VAS scores of patients in SGB+GA group at 2, 6, 12, and 24 h post-surgery were consistently lower than those in the control group (see Figure 2), with the differences being statistically significant (*P* < 0.05) (see Table 3).

**TABLE 3:** Comparison of postoperative pain scores between the 2 groups (*n* = 30).

Characteristic	Group		*p*-value	Effect size
	**GA-only, *N* = 30^1^**	**SGB+GA, *N* = 30^1^**		
2 h postop	4.00 (3.00, 5.00)	2.50 (2.00, 3.00)	< 0.001^2^	−0.802^3^
6 h postop	6.00 (4.25, 6.00)	3.00 (3.00, 4.00)	< 0.001^2^	−0.892^3^
12 h postop	6.00 (5.00, 7.75)	4.00 (3.00, 4.00)	< 0.001^2^	−0.689^3^
24 h postop	8.00 (6.00, 9.00)	4.00 (4.00, 5.00)	< 0.001^2^	−**0.922**^3^

^1^Median (IQR).

^2^Wilcoxon rank sum test.

^3^Cliff’s delta. Bolded value indicates statistically significant differences (i.e., *P* < 0.05).

### 3.4 Comparison of analgesic drug usage

The analysis of opioid consumption revealed significant intergroup differences (*P* < 0.001 for all comparisons). Patients receiving SGB+GA required 24.1% less intraoperative remifentanil compared to GA-only controls. Postoperatively, the SGB+GA group demonstrated a 75% reduction in median tramadol consumption. Notably, 40% of patients in the SGB+GA group required no tramadol rescue analgesia during the first 24 h, whereas all patients in the GA-only group (100%) needed supplemental doses (χ^2^ = 15.43) (see Table 4).

**TABLE 4:** Comparison of opioid consumption between groups.

Parameter	GA-only group (*n* = 30)	SGB+GA group (*n* = 30)	Statistic	*P*-value	Effect size
Total intraoperative remifentanil consumption (μg)	1,053.4 ± 210.8	789.2 ± 158.3	*t* = 5.67	< 0.001	1.42^1^
Postoperative 24-h tramadol consumption (mg)	200.0 (150.0, 250.0)	50.0 (0.0, 100.0)	*Z* = 4.35	< 0.001	0.65^2^
Patients requiring no tramadol rescue (%)	0.0 (0/30)	40.0 (12/30)	χ^2^ = 15.43	< 0.001	1.12^3^

^1^Cohen’s d.

^2^Cliff’s Delta.

^3^Cohen’s h. The symbol “*t*” denotes the result of the *t*-test, which, together with the corresponding *p*-value (<0.001), demonstrates a highly significant statistical difference in the “total intraoperative remifentanil consumption” metric between the two groups.

### 3.5 Occurrence of postoperative complications

The incidence of postoperative nausea and vomiting, dizziness, and drowsiness in SGB+GA group was lower than that in GA-only group, and the difference was statistically significant (*P* < 0.05). However, there was no statistically significant difference in the incidence of respiratory depression between the two groups (*P* > 0.05) (see Table 5).

**TABLE 5:** Comparison of postoperative complications between the 2 groups (*n* = 30).

Characteristic	Group		*p*-value	Effect size
	GA-only, *N* = 30^1^	SGB+GA, *N* = 30^1^		
Nausea and vomiting			0.028^2^	−0.53^4^
N	20 (66.7%)	27 (90.0%)		
Y	10 (33.3%)	3 (10.0%)		
Dizziness			0.038^2^	−0.49^4^
N	22 (73.3%)	28 (93.3%)		
Y	8 (26.7%)	2 (6.7%)		
Somnolence			0.103^3^	−0.48^4^
N	24 (80.0%)	29 (96.7%)		
Y	6 (20.0%)	1 (3.3%)		
Respiratory depression			> 0.999^3^	−0.36*
N	29 (96.7%)	30 (100.0%)		
Y	1 (3.3%)	0 (0.0%)		

^1^n (%).

^2^Pearson’s chi-squared test.

^3^Fisher’s exact test.

^4^Cohen’s d.

*Haldane correction used for respiratory depression.

### 3.6 Hospital stay

Patients in SGB+GA Group had a significantly shorter hospital stay compared to the control group (see Table 6).

**TABLE 6:** Comparison of length of hospitalization between the 2 groups.

Characteristic	Group		*p*-value	Effect size
	**GA-only, *N* = 30^1^**	**SGB+GA, **N** = 30^1^**		
Length of hospitalization	7.00 (7.00, 9.00)	5.00 (4.00, 6.00)	< 0.001^2^	−1.35^3^

^1^Median (IQR).

^2^Wilcoxon rank sum test.

^3^Robust Cohen.

^1^Rebound pain: Severe acute pain occurring after resolution of peripheral nerve blockade; Horner’s syndrome: transient triad of miosis, ptosis, and facial anhidrosis indicating successful sympathetic blockade.

## 4 Discussion

Shoulder arthroscopic surgery, known for its minimally invasive approach, rapid recovery, and low complication rate, has become the preferred surgical method for treating shoulder disorders (11). However, hemodynamic instability frequently occurs during these procedures due to surgical stimulation and pain (12). Our findings demonstrate that supplementing general anesthesia with a stellate ganglion block significantly attenuated fluctuations in mean arterial pressure and heart rate. This stabilizing effect can be attributed to the ability of SGB to suppress sympathetic nerve overactivity, promote vasodilation, and reduce peripheral vascular resistance, thereby promoting a more balanced cerebral oxygen supply and demand (13). This suggests that the integration of SGB effectively mitigates the surgical stress response and fosters superior hemodynamic maintenance throughout the operation.

Beyond stabilizing intraoperative hemodynamics, SGB exerted a profound impact on the quality of postoperative recovery, primarily through enhanced analgesia. Postoperative pain following shoulder arthroscopy is often dominated by a dull, movement-evoked character, arising from persistent sympathetic hyperactivity and neuroinflammation—elements that are notoriously poorly controlled by conventional peripheral nerve blocks (14). While techniques like the interscalene block provide excellent transient sensory blockade, their resolution can be accompanied by significant rebound pain driven by unresolved inflammatory cascades. Our findings are consistent with those of Uppal et al. (15), who also reported a substantial reduction in postoperative pain scores (approximately 3 points on the VAS at 24 h) following the implementation of a similar analgesic strategy. SGB circumvents this limitation via a multimodal, dual-pathway mechanism. Its selective sympathetic inhibition reduces norepinephrine release, dilates arterioles, and improves microcirculation (16), which in turn attenuates ischemic pain and accelerates the clearance of pro-inflammatory cytokines such as IL-6 and TNF-α (13, 16). These effects collectively blunt central sensitization and suppress pain amplification, which is particularly crucial during shoulder mobilization when tissue tension exacerbates neuroinflammatory signaling (17). Consequently, SGB delivered sustained analgesia that extended beyond 24 h without the characteristic rebound pain, thereby lowering immediate opioid requirements and permitting earlier and less painful engagement in rehabilitation—a key determinant of functional recovery. This aligns with clinical evidence underscoring the mechanistic advantages of sympathetic blockade (18, 19). The magnitude of pain reduction observed in our SGB+GA group not only confirms its efficacy against general anesthesia alone but also suggests a potential advantage over historical reports for brachial plexus blocks (20, 21), indicating that SGB offers a superior, multi-target strategy for managing complex postoperative pain by simultaneously inhibiting sympathetic activity and modulating inflammatory pathways.

The superior analgesia provided by SGB had direct and favorable downstream consequences. The significantly lower consumption of both intraoperative remifentanil and postoperative tramadol in the SGB+GA group provides a clear pharmacological basis for the observed reduction in opioid-induced complications. As nausea, vomiting, and dizziness are frequently driven by opioid use (22), the opioid-sparing effect of SGB—achieved through its dual inhibition of nociceptive transmission and inflammatory cascades (13, 16)—directly translated into a significantly lower incidence of these adverse events.

Ultimately, the synergistic benefits of improved hemodynamic stability, superior analgesia, and fewer complications converged to significantly shorten the hospital stay. Dull pain is a major impediment to active participation in physiotherapy, such as passive range-of-motion exercises. By effectively alleviating this type of pain, SGB enables patients to engage in rehabilitation sooner, accelerating key milestones like independent ambulation and self-care. This accelerated functional recovery is further potentiated by SGB’s opioid-sparing effects, as reduced opioid exposure minimizes sedation and gastrointestinal dysfunction, thereby facilitating earlier mobilization (23). Furthermore, enhanced microcirculation secondary to sympatholysis may also contribute to improved wound healing and a reduced risk of secondary complications like adhesive capsulitis, further expediting the overall recovery timeline.

From a healthcare economics perspective, a reduced length of stay (LOS) decreases direct medical costs and indirect burdens. Our data indicate that SGB could enhance bed turnover rates, a metric with significant implications for resource-constrained environments.

This study has several limitations. Its single-center retrospective design and relatively small sample size may limit the generalizability of the findings. Potential unmeasured confounding factors might also persist. Furthermore, the absence of a direct comparison with brachial plexus blocks, along with the reliance on subjective pain measures rather than objective biomarkers (e.g., IL-6) or multidimensional assessments, restricts a deeper mechanistic understanding of SGB’s therapeutic effects. The optimal dosage and concentration of local anesthetics for SGB also require further investigation. Future research should prioritize multicenter, prospective, three-arm trials incorporating dynamic monitoring of inflammatory biomarkers, standardization of procedural and follow-up protocols, and inclusion of 3-month functional outcomes to comprehensively evaluate the efficacy and recovery impact of SGB.

## 5 Conclusion

In shoulder arthroscopic surgery, the combination of SGB with general anesthesia offers numerous benefits. It stabilizes intraoperative hemodynamics, substantially reduces opioid requirements, lowers postoperative pain, minimizes complications, and shortens hospital stays. This combination makes it a valuable option for anesthesia and analgesia, ultimately enhancing patient outcomes and satisfaction. However, the application of SGB should be tailored to each individual patient. A thorough assessment of the patient’s condition is essential to ensure its safe and effective use.

Future research should involve large-sample studies to confirm these results, long-term follow-up to evaluate enduring effects, and multidisciplinary collaboration. This approach will help optimize the use of SGB and enhance patient care.

## Data Availability

The raw data supporting the conclusions of this article will be made available by the authors, without undue reservation.
